# Profiling and tandem mass spectrometry analysis of aminoacylated phospholipids in *Bacillus subtilis*

**DOI:** 10.12688/f1000research.7842.2

**Published:** 2016-04-05

**Authors:** Metin Atila, Yu Luo

**Affiliations:** 1 Department of Biochemistry, University of Saskatchewan, Saskatoon, Canada

**Keywords:** Gram-positive, Antibiotic resistance, Membrane, Charge, Phospholipid, Teichoic acid, alanyl-phosphatidylglycerol, lysyl-phosphatidylglycerol, aminoacylated phospholipids, D-alanylation

## Abstract

Cationic modulation of the dominantly negative electrostatic structure of phospholipids plays an important role in bacterial response to changes in the environment. In addition to zwitterionic phosphatidylethanolamine, Gram-positive bacteria are also abundant in positively charged lysyl-phosphatidylglycerol. Increased amounts of both types of lipids render Gram-positive bacterial cells more resistant to cationic antibiotic peptides such as defensins.  Lysyl and alanyl-phosphatidylglycerol as well as alanyl-cardiolipin have also been studied by mass spectroscopy. Phospholipids modified by other amino acids have been discovered by chemical analysis of the lipid lysate but have yet to be studied by mass spectroscopy. We exploited the high sensitivity of modern mass spectroscopy in searching for substructures in complex mixtures to establish a sensitive and thorough screen for aminoacylated phospholipids. The search for deprotonated aminoacyl anions in lipid extracted from *Bacillus subtilis *strain 168 yielded strong evidence as well as relative abundance of aminoacyl-phosphatidylglycerols, which serves as a crude measure of the specificity of aminoacyl-phosphatidylglycerol synthase MprF. No aminoacyl-cardiolipin was found. More importantly, the second most abundant species in this category is D-alanyl-phosphatidylglycerol, suggesting a possible role in the D-alanylation pathway of wall- and lipo-teichoic acids.

## Introduction

In most bacteria, phospholipids are the dominant cell membrane component ^1^. The phosphate moieties in these lipid molecules dictate the overall negative nature of bacterial membranes. This electrostatic feature makes them susceptible to cationic antibiotic peptides such as defensins ^2– 4^. In response to environmental challenges, bacteria constantly change their membrane composition ^1, 5^. Incorporation of less saturated and shorter fatty acyl chains makes the membrane more fluidic ^1^. Gram-negative bacteria generally have a high concentration of zwitterionic phosphatidylethanolamine (PE) which masks this anionic surface feature ^1, 6^. In comparison, Gram-positive bacteria in general have much less PE ^1^. However, they are abundant in aminoacylated phosphatidylglycerol (aminoacyl-PG), especially L-lysyl-PG ^5, 7^. The pivotal protein for aminoacyl-PG biosynthesis from L-aminoacyl-tRNA and PG is the lysyl-PG synthase MprF (multiple peptide resistance factor) ^8^ which appears to have a broad range of specificity for L-aminoacyl-tRNAs ^9, 10^. The crystal structures of the cytoplasmic catalytic domains of two MprFs, with one specific for lysyl- the other for alanyl-PG biosynthesis, have recently been elucidated ^11^. The catalytic domains of the two MprF enzymes have a long tunnel for accommodating PG with the catalytic site located at the narrowest part of the tunnel ^11^. The primary and tertiary structures of MprF resemble that of FemX which catalyzes L-alanyl transfer from tRNA to a peptidoglycan precursor ^12^. Both proteins are potential targets for novel antibiotics. MprF of *Bacillus subtilis* over-expressed in *Escherichia coli* has been observed to synthesize both L-lysyl-PG and L-alanyl-PG in the presence of aminoacyl-t-RNA ^10^. We expect to find both lysyl- and alanyl-PGs in *B. subtilis* lipids.

In comparison to Gram-negative bacteria, Gram-positive bacteria have profoundly different cell-envelope structures; they lack the outer membrane, and the cell wall is usually much thicker, with multiple peptidoglycan layers. In addition to PE and aminoacyl-PG biosynthesis, which modulate bacterial surface charge, one constant signature of Gram-positive cell envelopes, however, is the presence of additional glycopolymers including peptidoglycan-attached wall-teichoic acids and lipid-anchored lipoteichoic acids. This type of cell surface polymer was discovered in the late 1950s ^13^. It carries multiple negative charges due to its phosphodiester bonds between repetitive glycerol or ribitol residues. Its association with the cell envelope is anchored by covalent attachment to either membrane glycolipids or peptidoglycan ^14– 16^. The most common modification of this biopolymer is D-alanine esterification ^13, 15, 17^, which is carried out by four proteins (DltA, DltB, DltC and DltD) coded by the *dlt* operon ^18^. This surface charge modulation by D-alanylation appears to have profound effects on the antigenicity of the bacteria and immune response of host cells ^2^. The D-alanyl carrier protein ligase DltA (~500 amino acid residues) ^18^ is an enzyme resembling the adenylation domains (also called AMP-forming domains) found in modular nonribosomal peptide synthetases ^19^. Its remote homologues include the acyl-coenzyme A synthetases and firefly luciferases ^20^. DltA catalyzes the ATP-driven adenylation of the carboxyl group of D-alanine and the transfer of the activated D-alanyl to the thiol group of 4’-phosphopantetheine which is covalently attached to a serine side chain of D-alanyl carrier protein DltC (~80 amino acid residues) ^18, 21, 22^. The functional role has not been firmly established for DltB (~400 amino acid residues), an integral membrane protein. DltD (~400 amino acid residues), a membrane-bound protein via a putative N-terminal transmembrane helix, appears to bind DltC and possibly catalyzes the final D-alanyl transfer from DltC to teichoic acid ^23^. We suspect that a D-alanylated lipid species may serve as the intermediate between cytosolic D-alanyl-DltC and lipo- and wall-teichoic acids on the outside of cell membrane.

Lipid profiling of aminoacyl-PG using mass spectroscopy has been reported recently for *E. coli* and *B. subtilis* ^24^. L-alanyl-PG has been found to be abundant in Gram-negative *Pseudonomas aeruginosa* ^25^, which has a MprF homolog specific for L-analyl-tRNA substrate ^10^. D-alanyl- and L-lysyl-cardiolipin (CL) have also been separated from *Vagococcus fluvialis* ^26, 27^. However, only lysyl-PG has been identified in *B. subtilis* lipid by mass spectrometry ^24^. Serine, glycine and ornithine-containing lipids are also known to exist in bacteria ^28^. Here we report a more thorough profiling of aminoacyl-PGs. We also established sensitive scans for lysyl-PE as well as alanyl-PE. Importantly, the second most abundant aminoacyl-PG, alanyl-PG, appeared to be D-alanyl-PG, implying a role in the D-alanylation pathway of wall- and lipo-teichoic acids.

## Materials and methods

*Bacterial strain and cell culture.* The BL21 (DE3) strain of *E. coli* was acquired from Novagen. Strain 168 of *B. subtilis* and its mprF-deficient mutant (BKE 08425) were acquired from Bacillus Genetic Stock Center (BGSC). Both types of cells were first plated on LB-agar media. A single colony was inoculated into 10 ml of LB media. After over-night incubation at 37°C and 220 rpm in an environmental shaker, it was transferred to 1 liter of LB media. When the cell culture just reached an optical density of ~2.0 at 600 nm, the cell pellet was collected by centrifugation at 5,500 rpm for 16 min in a Beckman JLA-8.1 rotor at 4°C.

*Lipid extraction.* HPLC-grade organic solvents (Fisher Scientific) are used throughout the experiment. The lipid extraction procedure was adapted to get maximal yield of aminoacylated lipids based on the protocol developed by Folch ^29^. The wet cell pellet was re-suspended in equal weight of distilled and deionized water. The lipid extraction was carried out at a room temperature of 21°C except that the cells were kept on ice. 1.8 ml of the cell suspension was transferred to a glass centrifuge tube. Addition of 4 ml chloroform and 2 ml of methanol was followed by vortexing for 1 minute. 2 ml of methanol was added followed by 1 minute of vortexing. 2 ml of buffer solution (0.1 M NaAc at pH 4.5) was added followed by 1 minute of vortexing. Then the tube was placed on a rocking incubator for 3 hours. After that, the phase separation was assisted by centrifugation at 1,300 rpm for 5 minutes with a Beckman Allegro X-22R centrifuge. The heavier chloroform-rich phase was transferred by a glass syringe to a second glass centrifuge tube. The water-rich phase in the first tube was further extracted three times. Each time, 2 ml of chloroform was added, the mixture vortexed, the phase separation assisted by centrifugation, and the chloroform-rich phase transferred to the second glass tube. The combined chloroform-rich phase (~10 ml) in the second tube was first washed by adding 1 ml DI water followed by vortexing for 5 seconds. The lighter water-rich phase was removed after centrifugation. Another wash and dehydration cycle with 1.0 ml 0.5 M NaCl followed. After vortexing and subsequent centrifugation at 1,300 rpm, the chloroform-rich phase was collected into a third tube. This final sample was placed in a heater at 30°C and dried in an argon stream for approximately 2–3 hours. The empty tube was weighted, and again after drying. Typically, approximately 5 mg of total lipids were obtained and dissolved in chloroform to a concentration of 4 mg/ml.

*Chemical syntheses of aminoacylated derivatives of PE* – Lipids of PE with fatty acyl chains 16:0–18:1 were acquired from Avanti Polar Lipids. Fluorenylmethyloxycarbonyl chloride (Fmoc)-protected L-alanine as well as Fmoc and t-Butyloxycarbonyl (Boc)-protected L-Lysine were purchased from Sigma-Aldrich. 10 ml of dichloromethane (DCM) was added into a round bottom flask on ice with continuous stirring. 0.014 mmol Fmoc-Ala or Fmoc-Lys-Boc (2.0 × equivalents) was dissolved in the solvent followed by the addition of 0.016 mmol (2.2 × equivalents) NN-Dicyclohexylcarbodiimide (DCC). The PE chloroform solution was washed with saturated sodium bicarbonate. Then 0.007 mmol (1 × equivalent) PE was added dropwise in 1 minute. After 5 minutes of incubation on ice, the reaction mixture was placed in a water bath at the room temperature of 21°C for 1–2 hours. The reaction mixture was filtered through a 100 ml glass filter with fritted disc and then washed first with 1.0 ml of saturated sodium bicarbonate and then 1.0 ml of 0.5% HCl. The organic phase was dried by rotary evaporation, and was redissolved in 50% piperidine in dimethylformamide for the deprotection of Fmoc. Deprotection of Fmoc was carried out at room temperature for 4 hours, followed by the double wash and drying procedure described above. The lysyl-PE product was dissolved in DCM containing 10–20% trifluoroacetic acid (TFA) and incubated at room temperature for 30 minutes to remove Boc protection. The final product was double washed, dried, and redissolved in chloroform for storage at -80°C.

*Lipid analysis by thin-layer chromatography.* A total volume of 15 μl of lipid samples (4 mg/ml) were spotted 1.5 cm above the bottom edge on 0.25 mm thick silica gel on plastic sheet (Millipore) cut to a size of 10 cm × 20 cm. Alternatively, 100 μl of lipid samples were spotted on 1.0 mm thick silica gel on a glass plate (Fluka). After drying over a heater set at 50°C for ~10 minutes, the TLC sheet/plate was placed into a TLC chamber pre-equilibrated with a mixed solvent of chloroform : methanol : water (65:25:4). After ~30 minutes, the TLC sheet/plate was removed from the TLC chamber and dried for 5 minutes at 50°C. The TLC sheet/plate was first stained by spraying 0.01% primuline (Sigma-Aldrich) solution in acetone : water (80:20), dried in air or with mild heating for ~5 minutes. The fluorescent image was recorded with a Syngene G:BOX system. The fluorescent bands on the thicker gel were lifted and extracted by 100 μl of chloroform in a glass tube. The gel debris was discarded after centrifugation at 1,300 rpm for 1 minute. The TLC sheet was stained again by 0.1% ninhydrin (Sigma-Aldrich) in acetone : acetic acid (100:1), dried in air for ~5 minutes and heated at 100°C until purple spots appeared in a few minutes. The visible light image was recorded with the Syngene system.

*Lipid profiling by mass spectroscopy.* The lipid samples were diluted by adding 9-fold volume of methanol to a concentration of 0.4 mg/ml (or 400 ppm) for direct infusion at a rate of 0.6 ml/hour to a SCIEX 4000 QTRAP mass spectrometer. Electrospray ionization was achieved at a temperature of 500°C and a pressure of 20 psi for curtain gas as well as ion source gas 1 and 2. The collision energy in the ion trap was set at +45 or -65 electronvolts in positive and negative mode, respectively. A total of 30 MCA cycles of ion counts (optional) were accumulated as the mass spectra of precursor scans and neutral loss scans. The SCIEX Analyst software (version 1.6) was used to acquire and export averaged mass spectra. MS spectra in the figures were generated by Mass++ software (version 2.7.3) ^30^ or Microsoft Excel.

*Tandem mass spectroscopy.* The targeted MS/MS spectra were first acquired using the SCIEX 4000 QTRAP system. The parameters were the same as those for the profiling. High-accuracy MS/MS spectra were later acquired using an Agilent Q-TOF 6550 system. Direct infusion was set at a slower rate of 0.1 ml/hour for the Q-TOF 6550 system.

*Alkaline hydrolysis of lipids* – All lipids from one extraction procedure from 0.9 g of wet cells, dissolved in ~0.6 ml chloroform, was partially hydrolyzed by adding 0.25 ml of methanol and 0.04 ml of 1.0 M NH _4_OH and incubating at 37°C for 90 minutes without stirring. A volume of 0.05 ml 1.0 M formic acid was added to the solution followed by 0.5 ml of water. The mixture was vortexed for a few seconds and gently shaken in hand to partially remove bubbles. Then the mixture was centrifuged at 1,300 rpm for 5 minutes. The top water-rich layer was collected into a glass beaker and thoroughly dried at 90°C. The residue was dissolved in 0.1 ml water.

*Alkaline hydrolysis of bacterial cells* – 1.5 ml of cells at early stationary phase with an OD _600_ value (optical density at 600 nm) of ~2.0, were centrifuged at 13,000 rpm for 5 minutes. The cell pellet was subjected to 3 rounds of washing with 1.0 ml of water and centrifugation to discard the wash. The cells were deactivated by heating in boiling water bath for 10 minutes. Then the cells were suspended in 0.1 ml of 1.0 M NH _4_OH. The sample was incubated at 37°C for 90 minutes without stirring. The supernatant after centrifugation at 13,000 rpm for 5 minutes was transferred to a glass beaker and thoroughly dried (1 minute). The residue was dissolved in 0.1 ml of water.

*Conjugation with Marfey’s reagent* – 0.1 ml of 2 mM L- or D-alanine, or the samples from alkaline hydrolysis, was transferred to a glass vial with cap. Then 0.2 ml acetone, 0.05 ml 1% (~30 mM) Marfey’s reagent ^31^ in acetone, and 0.04 ml 1.0 M NaHCO _3_ were added and mixed by gentle shaking. The reaction solution was kept at 37°C for 90 minutes without stirring. As the reaction progressed, the bright yellow color of the solution turned into a darker color resembling maple syrup. The reaction was stopped by adding 0.05 ml 1.0 M formic acid.

*LC/MS analysis of* conjugated alanine – The reaction solution with Marfey’s reagent was diluted 10-fold into acetone. 2 μl of the diluted sample was injected into the LC system for inline LC/MS analysis using the SCIEX 4000 QTRAP system. An Agilent ZORBRAX Eclipse Plus C18 (2.1 mm × 100 mm) reverse-phase column was used. The aqueous solution A contained 10 mM NH _4_Ac at pH 4.6. The organic solution B is HPLC-grade acetone. The flow rate was set at 0.2 ml/minute. The gradient from 20% B to 80% B was developed in 10 minutes, followed by 2 extra minutes of column regeneration at 80% B and 8 minutes of equilibration at 20% B. The molecular anion of 340 amu corresponding to the alanyl-derivative of Marfey’s reagent was monitored by the mass spectrometer. The mixed alanine standard was a 1:1 mix of the diluted reaction solutions of D- and L-alanine.

## Results

MS scans in search for aminoacylated phospholipids and tandem mass spectra of aminoacylated phosphatidylglycerol and aminoacylated phosphatidylethanolamine The data sets are named as Figure 3B and such according to their appearance in the figures of the article.
Figure 2A: MS/MS spectrum of (30:0) lysyl-phosphatidylglycerol (lysyl-PG) anion (821 amu)
Figure 2B: MS/MS spectrum of (32:0) alanyl-PG anion (792 amu) 
Figure 3A: Precursor scan for 145 amu lysyl anion
Figure 3B: Precursor scan for 88 amu alanyl anion
Figure 3C: Precursor scan for 130 amu leucyl/isoleucyl anion
Figure 3D: Precursor scan for 132 amu aspartyl anion 
Figure 4A: Precursor scan for predominant 523 amu [DAG-OH]+ ion
Figure 4B: Scan for neutral loss of 269 amu head group of lysyl-phosphatidylethanolamine (lysyl-PE)
Figure 4C: Precursor scan for sodiated lysyl-PE head group of 292 amu
Figure 4D: Precursor scan for sodiated alanyl-PE head group of 235 amu 
Figure 5: MS/MS spectra of chemically synthesized (16:0-18:1) lysyl- and analyl-PE
Figure 5A: MS/MS spectrum protonated (16:0-18:1) lysyl-PE (846 amu)
Figure 5B: MS/MS spectrum of sodiated (16:0-18:1) lysyl-PE (868 amu)
Figure 5C: MS/MS spectrum of protonated (16:0-18:1) alanyl-PE (789 amu)
Figure 5D: MS/MS spectrum of sodiated (16:0-18:1) alanyl-PE (811 amu) 
Figure 6: High-accuracy MS/MS spectrum of (32:0) lysyl-PG (873 amu) 
Figure 7: High-accuracy MS/MS spectrum of (32:0) alanyl-PG (816 amu) 
Figure 9: Precursor scans for 88 amu alanyl anion and 266 amu sodiated alanyl-PG head group. Click here for additional data file. Copyright: © 2016 Atila M and Luo Y 2016 Data associated with the article are available under the terms of the Creative Commons Zero "No rights reserved" data waiver (CC0 1.0 Public domain dedication).

**Figure 1.**
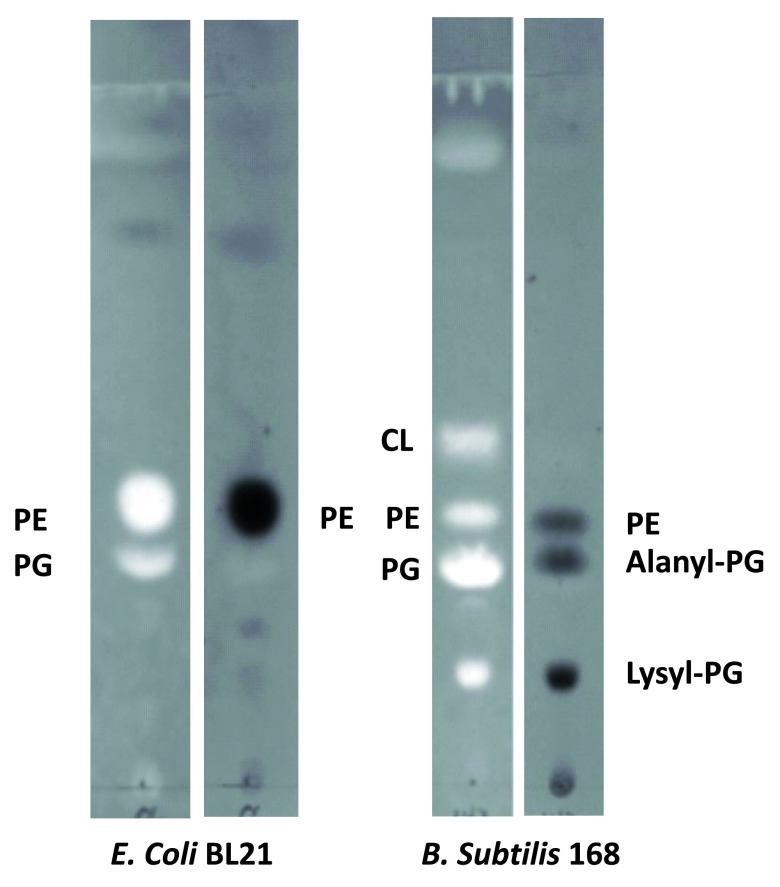
Thin-layer chromatogram of total lipids extracted from *E. coli* strain BL21(DE3) and *B. subtilis* strain 168. Major components of the bands are shown. **Bright panels:** Primuline-stained TLC sheet. **Dark panels:** Ninhydrin-stained TLC sheet. The fluorescent primuline (bright spots) is absorbed to hydrophobic molecules. Ninhydrin reacts with amino groups to produce purple products (dark spots). Lysyl-phospholipids form a distinct band with slow mobility. Alanyl-PG migrates slightly faster than PG, but significantly slower than PE. The Gram-negative *E. coli* has little CL.

*Profiling of major bacterial lipids* - We first modified the lipid extraction protocol using chloroform and methanol based on Folch method ^29^. Polar lipids from *E. coli* strain BL21(DE3) and *B. subtilis* strain 168 were extracted. Thin-layer chromatography was carried out to analyze the major components. Every primuline-stained major band on the TLC plate was collected using a razor and redissolved in 100 μl chloroform. We then acquired MS spectra of the total lipids as well as the TLC-separated lipids and tandem MS/MS spectra of dominant molecular ions. The major component of each primuline-stained band on the TLC plate was assigned ( Figure 1) based on the MS and MS/MS spectra. As expected, lipids extracted from *E. coli* strain BL21(DE3) were mainly composed of PE and PG ( Figure 1) ^6^. As expected, lipids from *B. subtilis* strain 168 were also rich in lysyl-PG and cardiolipin (CL) ( Figure 1). The relative abundance of PE was much lower in *B. subtilis* than *E. coli*. The most abundant ninhydrin-stained amino group-containing band was that of lysyl-PG. A third ninhydrin-stained band, which was visible in only a small fraction of lipid preparations, overlapped with the PG-rich band. The amino group in this band likely comes from alanyl-PG as indicated by mass spectroscopy. The ninhydrin-stained spot at the origin likely corresponds to free amino acids.

**Figure 2.**
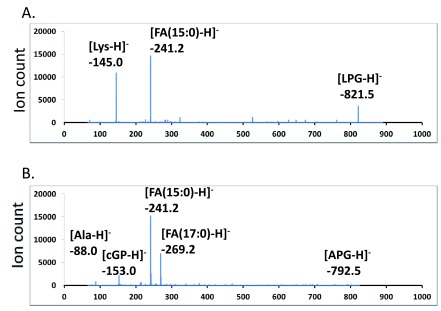
Tandem mass spectra of deprotonated lysyl-PG and alanyl-PG. Major peaks in the MS/MS spectra are labeled. FA – fatty acid, LPG – (30:0) lysyl-PG, APG – (32:0) alanyl-PG, cGP – cyclo-glycerol-phosphate. **A**. MS/MS spectrum of lysyl-PG. **B**. MS/MS spectrum of alanyl-PG.

*Tandem MS/MS spectra of lysyl- and alanyl-PGs* – The most abundant fragments of negative (deprotonated) molecular ions of phospholipids were fatty acyl anions ([FA-H] ^-^) of various sizes. Deprotonated molecular ions of PG and CL also dissociated to form a cyclo-glycerol-phosphate anion (153 amu), which is widely used to search for precursors that have a phosphoglycerol backbone ^32^. Lipids from the *E. coli* strain as the aminoacylated-PG-absent reference were richest in saturated tetradecanoic acid (14:0) and hexadecanoic acid (16:0) as well as (17:1) and (18:1) monounsaturated or cyclopropane fatty acids. This result was similar to the reported lipid composition in *E. coli* ^24^. The *B. subtilis* lipids, on the other hand, were most abundant in pentadecanoic acid (15:0) and heptadecanoic acid (17:0). The dominance of odd numbers of carbon atoms in the fatty acyl chain indicates that both bacteria likely utilize leucine or isoleucine primers for branch-chain fatty acid biosynthesis ^33^. The loss of a neutral head group from positive (protonated or sodiated) molecular ions of phospholipids produced dehydroxyl-diacylglycerol cations ([DAG-OH] ^+^) as the most abundant fragments. This neutral loss feature is commonly used to search for phospholipids with certain head groups ^34^. For instance, PE can be identified by a scan for the neutral loss of phosphoethanolamine (141 amu). We acquired tandem MS/MS spectra of lysyl- and alanyl-PGs in both positive and negative mode. The spectra in negative mode with a collision energy of -65 electronvolts were dominated by [FA-H] ^-^ and deprotonated aminoacyl ions: [Ala-H] ^-^ (88 amu) and [Lys-H] ^-^ (145 amu) ( Figure 2).

**Figure 3.**
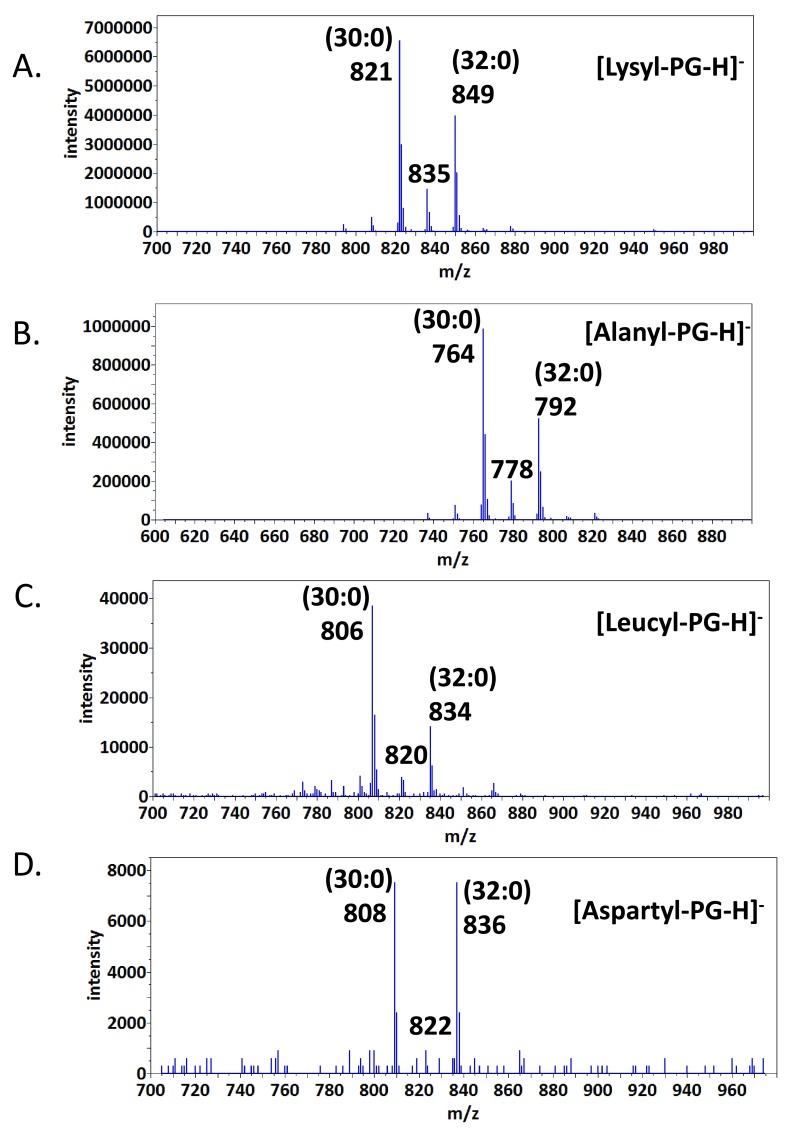
Precursor scans for aminoacyl-PGs. Major peaks in the spectra are labeled with fatty acid composition (number of carbon atoms: number of desaturation). **A**. Scan for precursors of a 145 amu anion. **B**. Scan for precursors of a 88 amu anion. **C**. Scan for precursors of a 130 amu anion. **D**. Scan for precursors of a 132 amu anion.

*Precursor scans for aminoacyl-PGs* – Besides lysyl- and alanyl-PGs, there were no molecular ions in the MS spectra which matched expected m/z values for other types of aminoacyl-PG ions. The abundance of the deprotonated aminoacyl ions prompted us to utilize this structural feature to search with high sensitivity for lipid precursors which produce such fragment ions. We first tested this protocol on lysyl- and alanyl-PGs and similarly esterized CL. The dominant molecular anions from the precursor scans at a collision energy of -65 electronvolts matched the expected m/z values of lysyl-PG and alanyl-PG species with two (15:0) or (17:0) fatty acyl chains ( Figure 3A & 3B). The two types of lipid species with less abundant fatty-acyl compositions were also identified as peaks separated by 14 amu that corresponds to a methylene group. No aminoacyl-CL was identified in higher mass range around 1500 amu. We then applied such precursor scans to search for other aminoacylated PGs or CLs. With the exception of cysteine, the scans identified correct sized candidates of molecular anions of 17 aminoacyl-PGs with two clearest examples of leucyl-PG and aspartyl-PG shown in Figure 3C & 3D. The scans were not able to differentiate between glutamine and lysine, or between leucine and isoleucine, which share similar or identical molecular mass.

**Figure 4.**
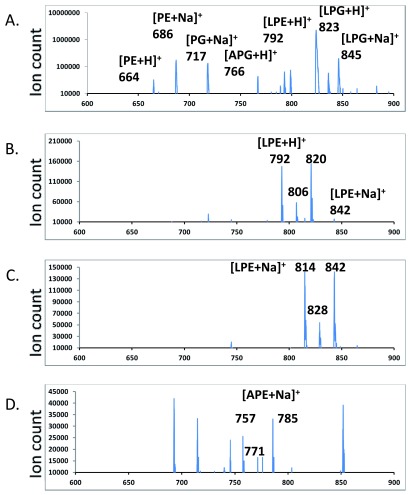
Neutral loss and precursor scans for aminoacyl-PEs. Major peaks in the MS/MS spectra are labeled. LPG – lysyl-PG, APG – alanyl-PG, LPE – lysyl-PE, APE – alanyl-PE. **A**. Scan for precursors of a 523.3 amu DAG fragment. **B**. Scan for a neutral loss of a 269.1 amu fragment. **C**. Scan for precursors of a 292.1 amu fragment. **D**. Scan for precursors of a 235.1 amu fragment.

**Figure 5.**
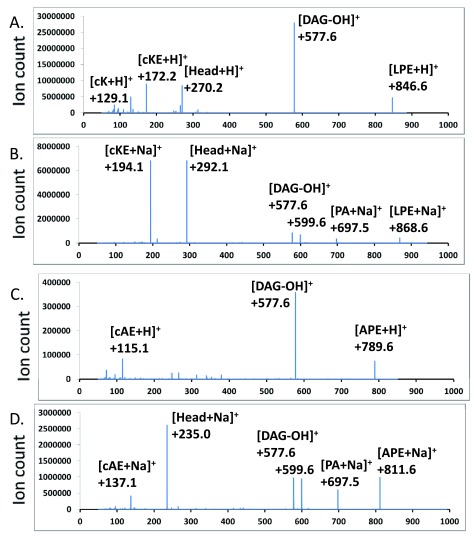
MS/MS spectra of chemically synthesized lysyl-PE and alanyl-PE. Protonated and sodiated ions are shown. LPE – (16:0–18:1) lysyl-PE, APE – (16:0–18:1) alanyl-PE, PA – phosphatidyl acid, DAG – diacylglycerol, Head – head group, cAE – cyclo-alanyl-ethanolamine, cK – cyclo-lysine, cKE – cyclo-lysyl-ethanolamine. **A** & **B**. Lysyl-PE. **C** & **D**. Alanyl-PE.

*Neutral loss and precursor scans for aminoacyl-PEs* – There were no major peaks which corresponded to molecular ions of aminoacyl-PEs. We first employed a scan at an optimized collision energy of +45 electronvolts that searches for precursors of the most abundant [(30:0) DAG-OH] ^+^ fragment ion (523 amu). The major hits corresponded to protonated as well as sodiated PE, PG, lysyl-PG, alanyl-PG as well as several other less abundant species including one at 792 amu which corresponded to the expected size of a protonated lysyl-PE species ( Figure 4A). No other ions matched expected m/z values of aminoacyl-PEs. We then employed scans for molecular cations, also at +45 electronvolts, which produced fragments that resulted from the neutral loss of head groups (269 amu for lysyl-phosphoethanolamine, 212 amu for alanyl-phosphoethanolamine). The scan for the neutral loss of 269 amu found strong candidates for lysyl-PEs ( Figure 4B). It is worth noting that their putative fatty acyl compositions (30:0, 31:0, 32:0) matched those of the dominant molecular ions of PE, PG, lysyl-PG and alanyl-PG. We then synthesized (16:0–18:1) lysyl-PE and alanyl-PE. The tandem MS/MS spectra of chemically synthesized lysyl-PE and alanyl-PE were also acquired ( Figure 5). In addition to the major fragment [DAG-OH] ^+^ ion, the sodiated molecular cations also dissociate to produce intense fragment peaks which corresponded to the sizes of the sodiated head groups (292 amu and 235 amu, respectively). Additional scans at a collision energy of +45 electronvolts for precursors which generate such sodiated head group ions revealed hits which were in agreement with the neutral loss scan for lysyl-PE ( Figure 4B & 4C), and implied the existence of alanyl-PE (757, 771 and 785 amu ions in Figure 4D).

*Irreproducibility of aminoacyl-PEs* – Although the presence of aminoacyl-PEs was interesting at first, we no longer found their presence when several new batches of the bacterial polar lipids were extracted without the final drying step at 30°C. It appears that the drying process may have caused existing species PE and aminoacyl-PGs in the lipids to chemically react to produce aminoacyl-PEs. This should be a caution which needs attention while sensitive profiling by mass spectrometry is employed.

**Figure 6.**
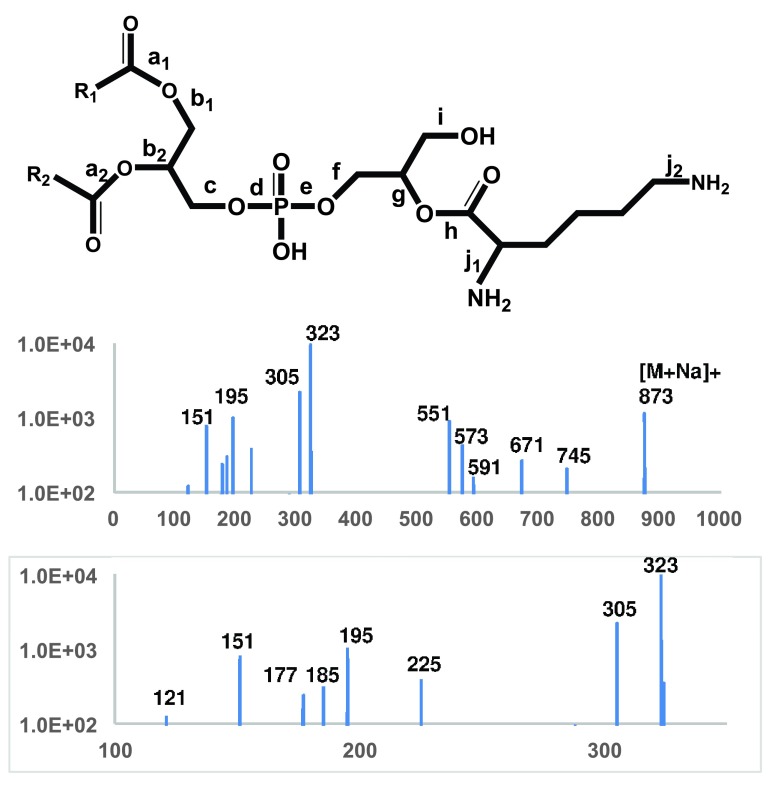
Tandem MS spectrum of (32:0) lysyl-PG. MS/MS spectrum of sodiated lysyl-PG (873 amu) ions is shown. The molecular structure is shown on the top with scissile bonds labeled alphabetically. The horizontal axis represents m/z values. The vertical axis represents ion counts.

*Tandem mass spectra of L-lysyl-PG* – The 4000 QTRAP system for liquid profiling has a practical resolution of 0.7 amu and accuracy of 0.2 amu. The Q-TOF 6550 system, optimized for proteomic research in positive mode, was tuned to reach a much higher accuracy of ~2 ppm or within 0.001 amu. We acquired high-accuracy MS/MS spectra of putative molecular ions of lysyl- and alanyl-PGs to further verify our assignments and to obtain clues for devising sensitive scans for lipid profiling. There were only two aminoacyl-PGs, lysyl-PG and alanyl-PG, that produced abundant enough (over 1000 counts) molecular ions for tandem mass spectrometry study. In fact, lysyl-PG was expectedly one of the most abundant phospholipids in the bacterium. It is certainly L-lysyl-PG as its lysyl group is known to have originated from L-lysyl-tRNA. In negative mode with collision energy set at -50 electronvolts, lysyl-PG ions of 821 and 849 amu produced an abundance of deprotonated lysyl ions besides two major – (15:0) and (17:0) - fatty acyl anions at 241 and 269 amu. The observed mass of [Lys-H] ^-^ was 145.0972, matching the calculated monoisotopic mass of 145.0978. A deprotonated glutamine ion would have a distinctively different mass of 145.0613. We also acquired MS/MS spectrum of sodiated (32:0) lysyl-PG cation (873 amu) at a collision energy of +40 electronvolts ( Figure 6). Although protonated lysyl-PG ions were also abundant (823 amu and 851 amu), they produced less prominent fragments than the sodiated ions. Since a sodiated lysyl-PG but not a protonated lysyl-PG has a potent neutral amino group for intramolecular nucleophilic substitution, it is not surprising that we observed plenty of prominent structural features ( Table 1) from the sodiated lysyl-PG ion (873 amu). For instance, it produced sodiated cyclo-lysine (151.0841 amu vs a calculated mass of 151.0848 amu) to verify the presence of lysyl residue along with the deprotonated lysyl ion in negative mode. It produced cyclo-lysyl-glycerol in both protonated (185.1275 vs 185.1291) and sodiated form (225.1205 vs 225.1216) to further indicate that the lysyl residue is attached to the glycerol head group. The fragments of sodiated cyclo-lysyl-glycerolphosphate (305.0868 vs 305.0879) and lysyl-glycerolphosphate (323.0972 vs 323.0985) finally completed the head group assignment. The outstanding abundance of the 323 amu fragment made a precursor scan for this fragment in positive mode the second best behind the precursor scan for deprotonated lysyl ion (145 amu) in negative mode. Both protonated and sodiated dehydrated DAG fragments were abundant (551 and 573 amu, respectively). So was sodiated DAG (591 amu). The neutral loss of the cyclo-lysyl-glycerol head group (202 amu) produced sodiated phosphatidic acid (671 amu) and that of cyclo-lysine (128 amu) produced sodiated PG (745 amu). It is worth noting that neutral losses of fatty acid (RCOOH) or ketene (R-C=C=O) produced minor peaks less than 100 counts, which are not shown in Figure 6.

**Table 1.** Accurate masses of fragments from lysyl-PG.

Observed mass	Calculated Mass	Cleavage	Description
120.9655	120.9667	c & f	Pho + Na ^+^
151.0841	151.0848	h	cyclo-Lys + Na ^+^
176.9913	176.9929	c & g	cyclo-Gro-Pho + Na ^+^
185.1275	185.1291	f & I	cyclol-Lys-Gro - OH
195.0022	195.0035	c & h	Gro-Pho + Na ^+^
225.1205	225.1216	f	cyclo-Lys-Gro + Na ^+^
305.0868	305.0879	d	cyclo-Lys-Gro-Pho + Na ^+^
323.0972	323.0985	c	Lys-Gro-Pho + Na ^+^
551.5018	551.5040	c	DAG - OH
573.4839	573.4862	c	DAG -H _2_O + Na ^+^
591.4942	591.4968	d	DAG + Na ^+^
671.4604	671.4631	f	PA + Na ^+^
745.4967	745.4999	h	PG + Na ^+^

Note: The alphabetically labeled scissile bonds are shown in Figure 6 and Figure 7. PA – phosphatidic acid; PG – phosphatidylglycerol; DAG – diacylglycerol; Pho – phosphate; Lys – lysine; Gro – glycerol. A cyclic compound in mass is equivalent to a dehydrated compound.

**Figure 7.**
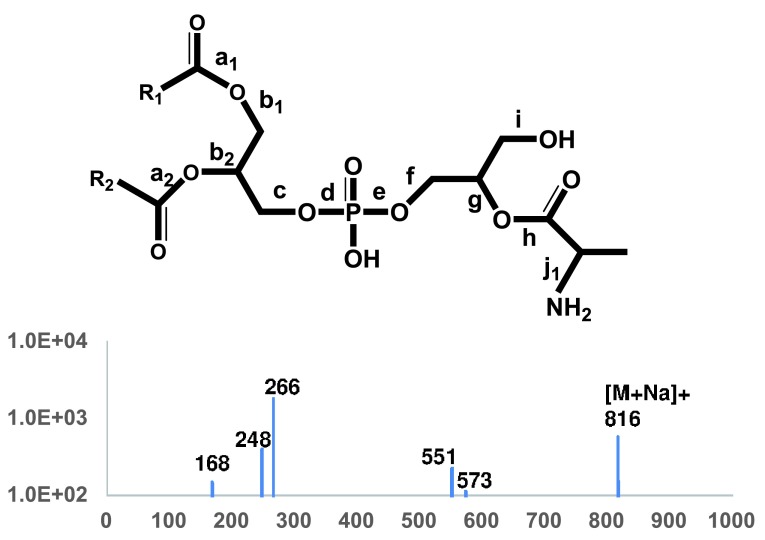
Tandem MS spectrum of (32:0) alanyl-PG. MS/MS spectrum of sodiated alanyl-PG (816 amu) ions is shown. The molecular structure is shown on the top with scissile bonds labeled alphabetically. The horizontal axis represents m/z values. The vertical axis represents ion counts.

*Tandem mass spectra of alanyl-PG* – Since alanyl-PG appeared to be much less abundant than lysyl-PG, we first chose the lipid preparation with the highest abundance of alanyl-PG as revealed by TLC analysis ( Figure 1) for tandem mass spectra acquisition. The most abundant putative alanyl-PG anions were observed at 764 and 792 amu, corresponding to (30:0) and (32:0) alanyl-PG, respectively. Besides the fatty acyl anions, they produced an abundance of alanyl anion at 88.0396 amu, closely matching expected value of 88.0399. Fragmentation at putative protonated alanyl-PG ions (766 and 794 amu) did not produce meaningful results. We conclude that they are not abundant enough for tandem MS analysis. The sodiated alanyl-PG cation (816 amu), corresponding to (32:0) alanyl-PG, was abundant enough to produce a simpler MS/MS spectrum ( Figure 7 and Table 2) than that of its lysyl-PG counter-part ( Figure 6 and Table 1). The presence of the 168 amu ion was critical for the assignment of the alanyl-glycerol attachment, as it corresponds to sodiated cyclo-alanyl-glycerol (168.0623 vs 168.0640). The presence of the whole alanyl-glycerolphosphate head group was verified by the presence of sodiated cyclo-alanyl-glycerolphosphate (248.0287 vs 248.0300) and sodiated alanyl-glycerolphosphate (266.0395 vs 266.0410). In fact, the outstanding abundance of the 266 amu cation makes a precursor scan for this fragment the second most sensitive lipid profiling scan for alanyl-PG behind the scan for deprotonated alanine (88 amu). As for lysyl-PG, the DAG residues were also abundant (551 and 573 amu).

**Table 2.** Accurate masses of fragments from alanyl-PG.

Observed mass	Calculated mass	Cleavage	Description
168.0623	168.0640	f	cyclo-Ala-Gro + Na ^+^
248.0287	248.0300	d	cyclo-Ala-Gro-Pho+ Na ^+^
266.0395	266.0410	c	Ala-Gro-Pho + Na ^+^
551.5018	551.5040	c	DAG - OH
573.4839	573.4860	c	DAG - H _2_O + Na ^+^

Note: The alphabetically labeled scissile bonds are shown in Figure 6 and Figure 7. The abbreviation are the same as in Table 1. Ala - alanine.

**Figure 8.**
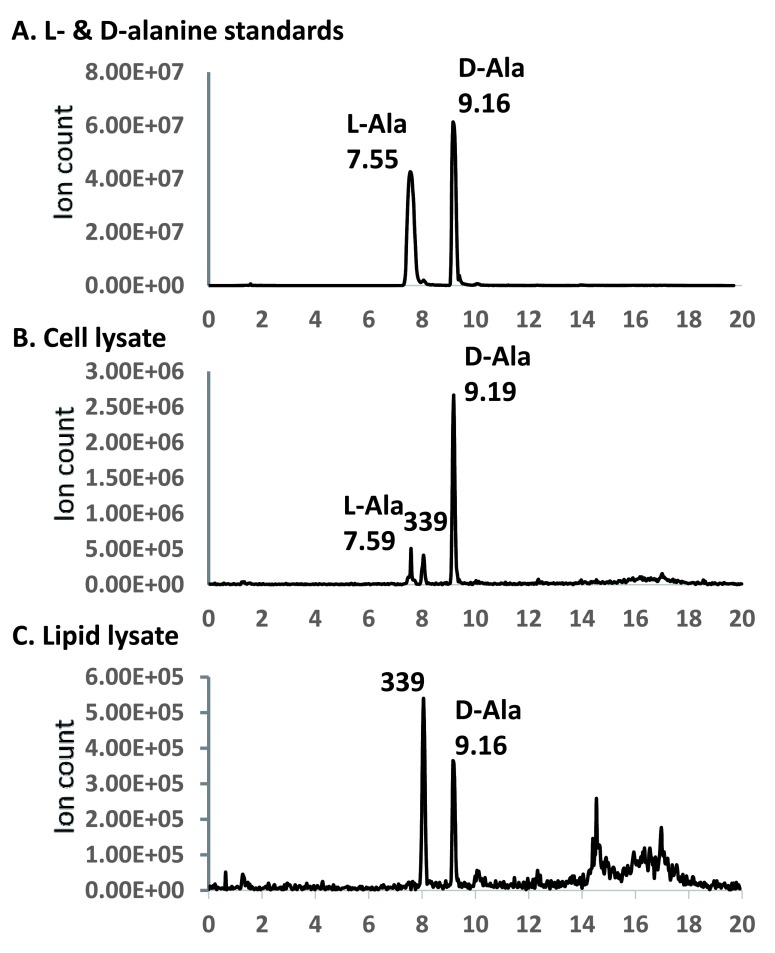
LC/MS chromatograms of alanine in cell and lipid lysates. The 340 amu molecular anion was monitored. The horizontal axis corresponds to retention time (minute). Peak retention times are marked. The peaks at 8.05 minutes, which correspond to a background 339 amu anion, is marked with “339”.

*LC/MS analysis of D- and L-alanine in lipid and whole cell lysates* – Alanyl groups in the bacterial lipids as well as on bacterial cell surface are known to be labile under mild alkaline conditions ^35, 36^. We established a set of alkaline hydrolysis and alanine extraction protocols that were practically complete at both hydrolysis and extraction stages. Since ammonia and formic acid residues were removed by evaporation, these reagents did not pose any interference with later experimental steps of TLC, conjugation with Marfey’s reagent, and mass spectrometry. By Marfey’s design, the derivatives of D-amino acids tend to have longer retention times on a reverse-phase column than their respective L-amino acid derivatives. The D-alanyl-derivative of Marfey’s reagent eluted significantly later and as a higher and sharper peak at 9.16 minutes than that of the L-alanyl-derivative which eluted at 7.55 minutes ( Figure 8). The alanine released by alkaline hydrolysis of bacterial cells was predominantly D-alanine (~90%), while that of lipids was exclusively D-alanine. By comparing the ion counts with the standard D- and L-alanine solution (equivalent to 1 mM each), the 0.1 ml lysate from 1.5 ml of bacterial cells contained ~0.05 mM D-alanine, while the 0.1 ml lipid lysate contained ~0.005 mM D-alanine. Considering that the lipid lysate was derived from ~100-fold more bacterial cells than the whole cell sample, there appeared to be 1000-fold more D-alanine on the cell surface than that in the membrane. We typically obtained 5–10 mg dried lipids from 0.9 g wet cell pellet, which corresponds to a weight ratio of approximately 100. By taking into account this weight ratio, we estimate that whole cell still has ~10-fold denser D-alanine than membrane. Importantly, the alanylated phosphatidylglycerol is D-alanyl-PG, it is therefore not synthesized from tRNA-carried L-alanyl by a reaction catalyzed by MprF. Instead, another well-known source of activated alanine carried by D-alanine carrier protein DltC in the form of thioester ^22, 37^ may be the most likely origin.

**Figure 9.**
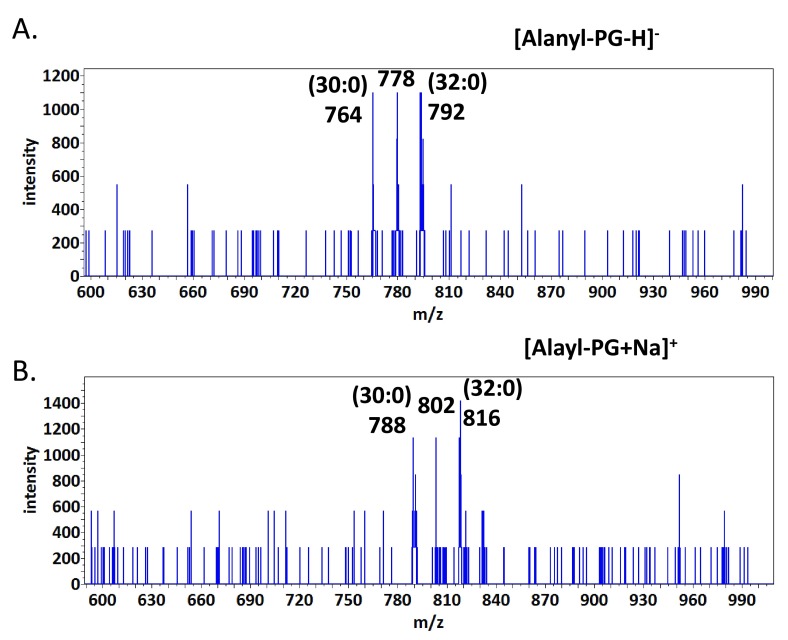
Precursor scans for alanyl-PGs in lipids extracted from the mprF-deficient strain. Major peaks in the spectra are labeled with fatty acid composition (number of carbon atoms: number of desaturation). **A**. Scan for precursors of a 88 amu anion. **B**. Scan for precursors of a 266 amu cation. No MCA cycles were employed.

*Precursor scans for aminoacyl-PGs in lipids extracted from the mprF-deficient strain* – We later extracted lipids from the mprF-deficient strain and did lipid profiling analysis. The lipids did not show the presence of lysyl-PG based on precursor scans for 145 amu lysyl anion and 323/301 amu sodiated/protonated lysl-glycerolphosphate head group. We also acquired precursor scan spectra for 283 amu dehydrated and protonated head group of lysyl-PG and its 266 amu residue due to the neutral loss of ammonia. As expected for the mprF mutant, Lysyl-PG was not detected. On the other hand, precursor scans for 88 amu alanyl anion and sodiated alanyl-glycerol-phosphate head group both indicated the presence of alanyl-PG ( Figure 9) even without 30 cycles of signal propagation. Tandem mass spectrometry analysis confirmed the identity of the 764 amu precursor anion as (30:0) alanyl-PG, and the identity of the 788 amu cation as sodiated (30:0) alanyl-PG.

## Discussion

Aminoacylated lipids play an important role in regulating the surface charge of Gram-positive bacteria ^5^. It appears that mass spectrometry can be exploited to successfully search for trace amounts of aminoacylated phospholipids. Mass spectrometry also makes identification of known as well as unknown lipids possible even without separation or chemical synthesis. The positive results on a broad range of aminoacylated-PGs are consistent with previous work on lipid hydrolysate ^7^. The intensities of various aminoacyl anions dissociated from the bacterial lipids span at least 3 orders of magnitude with lysyl anion being the strongest (6 × 10 ^6^) followed by alanyl (9 × 10 ^5^), leucyl/isoleucyl (4 × 10 ^4^) and aspartyl (7 × 10 ^3^) ( Figure 3). It is worth noting that the latter two molecular ions did not show appreciable peaks in the MS spectrum. The aminoacyl-PG synthase MprF is known to have a broad range of aminoacyl-tRNA specificity ^10^. Our results may have provided a semi-quantitative measure of the specificity of *B. subtilis* MprF.

Since PE is a major component of bacterial lipids, we also searched for aminoacylated derivatives of PE. The amide-linked PE derivative cannot be identified by their PG counterparts’ dissociation into deprotonated aminoacyl ions. We therefore employed the neutral loss (NL) scanning methodology based on those commonly used for identifying phosphatidylethanolamine (NL of 141 amu head group), phosphatidylserine (NL of 185 amu head group), phosphatitylacid (NL of 115 amu ammoniated head group) and phosphatidylinositol (NL of 277 amu ammoniated head group) ^34^. We also searched for precursors of the most abundant dehydroxyl-diacylglycerol cation (523 amu) which has the dominant fatty acyl composition of (30:0). The resulting spectrum provided a representative survey of all major species of phospholipids ( Figure 4A). MS/MS spectra of chemically synthesized lysyl-PE and alanyl-PE revealed intense peaks corresponding to sodiated head groups, which led to high-sensitivity precursor scans. The sodium ion appeared to have played an important role in generating intense peaks of head group fragments as well as contributing to high yield in lipid extraction due to its inert chemical property in comparison to commonly used ammonium salt. Other metal ions such as cesium which, like sodium, has only one stable isotope, can be further exploited for enhanced sensitivity in lipid profiling ^38^. Although the presence of lysyl- and alanyl-PEs appeared to be accidentally introduced in the lipid drying process, we did have established a sensitive enough lipid profiling method to rule out their biological relevance in *B. subtilis*.

**Figure 10.**
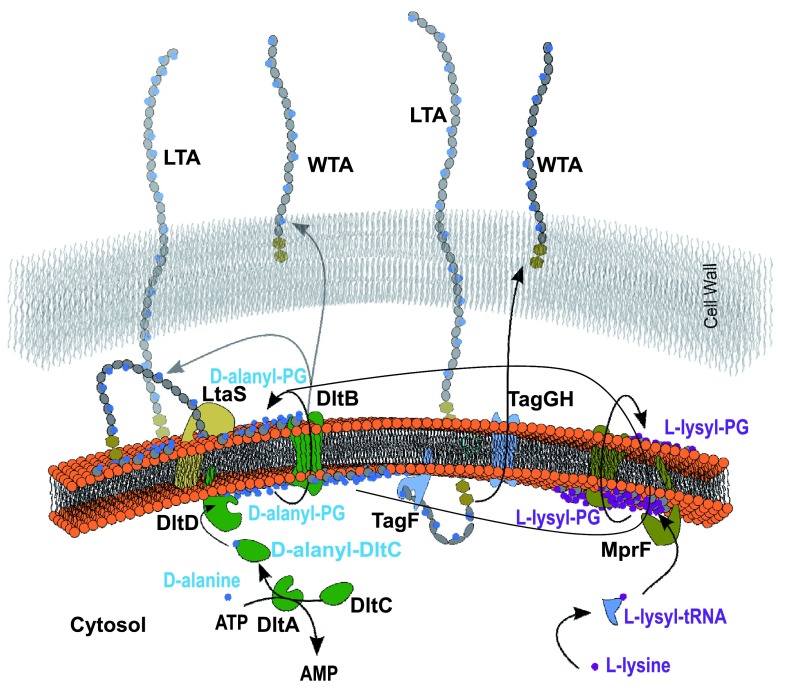
Possible D-alanylation pathways of lipo- and wall-teichoic acids. Arrows depict either transport or transfer processes. The dihexosyl parts of teichoic acids are shown as twin hexagons. The head group of phosphatidylglycerol and repeating glycerolphosphate units in teichoic acids are shown in grey circle and ellipse, respectively.

**Figure 11.**
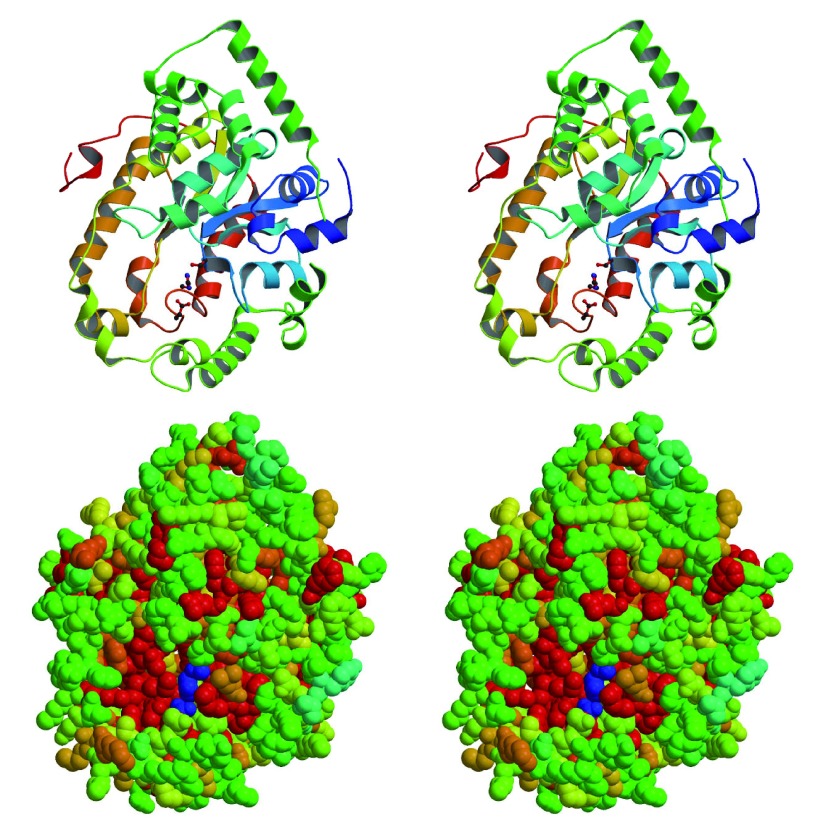
Structure of DltD from *Streptococcus pneumoniae* in stereo. **A**, Ribbons diagram of DltD. The side chains in the Ser-His-Asp triad are shown in ball-and-stick model. **B**, Space-filling model of DltD. The most conserved residues are shown in red, and the least conserved in green. The putative catalytic triad is shown in blue.

Importantly, the identification of D-alanyl-PG rather than L-alanyl-PG apparently rules out the relevance of the aminoacyl-tRNA-dependent MprF in its biosynthesis. The mprF-deficient strain also produced alanyl-PG. Instead, the *dlt* operon, which codes four proteins named sequentially as DltA-D, comes into focus. The cytosolic DltC protein serves as the alanyl carrier protein with a serine-attached 4’-phosphopantetheine as the site for alanyl-thioester formation in the presence of ATP and catalyzed by DltA. Biological functions of the two membrane-bound proteins DltB and DltD have yet to be fully characterized. Mysteriously, the targets of DltC-carried alanyl group are lipoteichoic acid located at the outer leaflet of cytoplasmic membrane, and wall-teichoic acid covalently attached to peptidoglycan. We have long suspected the presence of a D-alanylated lipid as an intermediate for the eventual transfer of D-alanine from the cytosol to lipoteichoic acid. D-alanyl-PG may just be this putative intermediate D-alanyl carrier. Figure 10 illustrates a list of possible pathways for the transfer of D-alanine to lipo- and wall-teichoic acids. First, the D-alanylated lipid may be produced by DltD, and transported to the outer leaflet by a flippase such as the integral membrane protein DltB or the pore domain of MprF which is known to transport L-lysyl-PG and other L-aminoacyl-PGs ^9^. Second, D-alanyl-PG can be transferred to lipo- and wall-teichoic acids by a transferase such as DltB, a putative membrane-bound O-acyltransferase ^39^, or incorporated as D-alanyl-glycerolphosphate units from D-alanyl-PG into the growing ends of teichoic acids by their respective polymerases LtaS ^40, 41^ and TagF ^42, 43^. It is worth noting that D-alanyl-CL has been reported before ^27^. As alanyl-PG and alanyl-CL share an ester bond with the glycerol head group, both are candidates for the lipid intermediate for D-alanylation of teichoic acids. Our hypothesis is also based on the best biochemical evidence, or the lack thereof, on DltB and DltD. DltD was previously observed to bind specifically to DltC and possess thioesterase activity on D-alanyl-acyl carrier protein ^23^. If we substitute the water nucleophile in the thioesterase-catalyzed reaction for hydroxyl in the head group of PG, DltD would become a D-alanyl transferase. In addition, our bioinformatics analysis of crystal structure of *Streptococcus pneumoniae* DltD (PDB entry 3BMA, deposited by New York SGX Research Center for Structural Genomics) using ProFunc ^44^ revealed a Ser-His-Asp triad embedded in a structure ( Figure 11) with overall similarity to platelet-activating factor acetylhydrolase (PDB code 1BWR), which belongs to the phospholipase A2 category. Apparently, the putative catalytic triad (Ser-47/Asp-376/His-379) in the *S. pneumoniae* DltD is conserved in all known DltD orthologs in Gram-positive bacteria. Since many phospholipid synthases belong to a superfamily of phospholipase D ^1^, a synthase in the superfamily of phospholipase A2 would not be surprising. We therefore hypothesize that DltD may serve as the synthase of D-alanyl-PG, which may serve as the key lipid D-alanyl carrier for the D-alanylation pathway of teichoic acids.

## Data availability

The data referenced by this article are under copyright with the following copyright statement: Copyright: © 2016 Atila M and Luo Y

Data associated with the article are available under the terms of the Creative Commons Zero "No rights reserved" data waiver (CC0 1.0 Public domain dedication). 

*F1000Research*: Dataset 1. MS scans in search for aminoacylated phospholipids and tandem mass spectra of aminoacylated phosphatidylglycerol and aminoacylated phosphatidylethanolamine, 10.5256/f1000research.7842.d117480 ^45^
