# Supplementary material for: Profiling and tandem mass spectrometry analysis of aminoacylated phospholipids in Bacillus subtilis 
Source: F1000Res. 2016 Apr 5;5:121. Originally published 2016 Jan 29. [Version 2] doi: 10.12688/f1000research.7842.2 (PMC4792211; doi:10.12688/f1000research.7842.2)
Supplement: Peer review history

# Peer review: Referee response for version 1

**DOI:** 10.5256/f1000research.8441.r12660

**Published:** 2016-03-14

**Authors:** Markus Ralser, Christoph Messner

**Affiliations:** 1 Cambridge Systems Biology Centre, University of Cambridge, Cambridge, UK; 2 University of Cambridge, Cambridge, UK

The manuscript by Atila and Luo presents a helpful, descriptive mass spectrometric analysis of the aminoacylated phospholipids in *Bacillus subtilis*. The authors acquired product ion scans of lysyl and alanyl phosphatidylglycerol, as well as precursor ion scans and neutral loss scans for different aminoacyl phosphatidylglycerols and aminoacyl phosphatidylethanolamine. They found 17 different aminoacyl phosphatidylglecerols but no aminoacyl phosphatidylethanolamine in the lipid extracts. Furthermore, D- and L- alanine were quantified after alkaline hydrolysis of the lipid extracts, and only the D enantiomer was found. The authors conclude that D-alanyl phosphatidylglycerol is present, which is a D-alanine carrier from the cytosol to lipoteichoic acid.

As already mentioned by the referee 1, the most interesting finding of this study is that alanyl phosphatidylglecerol is composed solely of the D enantiomer. However additional control measurements which support this finding are highly recommended. I support the suggestion of referee 1 to test if alanyl-PG is formed in MprF deficient mutants, other controls with chemical standards are possible as well.

In Figure 8, the ion count of D-alanine in the cell lysate seems much larger as in the lipid lysate; it is difficult to understand how this leads to the same concentration estimate  (0.05 mM). In general the assumption that “the cell has ~10 fold denser d-alanine than membrane” appears speculative and is based on several assumptions (linear response of the instrument, quantitative hydrolysis and extraction). I suggest to remove this part or to make a more elaborate analysis. Have the authors analysed alanyl PGs in the chloroform rich phase after hydrolysis to check the efficiency of the hydrolysis? Furthermore the authors could measure the concentrations of free alanine by applying the same protocols without adding NH _4_OH to check the efficiency of the hydrolysis step.

The authors reported an irreproducible measurement of aminoacyl PE, potentially caused by the drying process. How long did this 30C drying procedure take? The formation of aminoacyl PEs due to chemical reactions of PE and aminoacyl PGs would indicate an instability of the aminoacyl PGs. Therefore, how reproducible are the measurements of the aminoacyl PGs (variation in ion count)? Are these also influenced by the drying process?

The authors used collision energies of +45 and -65eV with the QTRAP MS and -40 and +50 eV with the Q-Tof system. Potential caveats resulting from the differently chosen fragmentation settings should be discussed.

What is the m/z of the precursor ion for the tandem MS measurements of deprotonated lysyl and alanyl PG (Figure 2)? m/z 821.5 and 792.5?

We suggest to remove figure 10, as it provides no relevant information in the context of the findings reported in this study.

We have read this submission. We believe that we have an appropriate level of expertise to confirm that it is of an acceptable scientific standard, however we have significant reservations, as outlined above.

# Response

**Published:** 2016-03-14

**Authors:** Yu Luo

**Affiliations:** Department of Biochemistry, University of Saskatchewan, Canada

*YL: Thank you Dr. Rasler for your comments. We will incorporate part of our response to your comments in the revised version. For your comments on the methodologies, our response is listed below.*

The manuscript by Atila and Luo presents a helpful, descriptive mass spectrometric analysis of the aminoacylated phospholipids in *Bacillus subtilis*. The authors acquired product ion scans of lysyl and alanyl phosphatidylglycerol, as well as precursor ion scans and neutral loss scans for different aminoacyl phosphatidylglycerols and aminoacyl phosphatidylethanolamine. They found 17 different aminoacyl phosphatidylglecerols but no aminoacyl phosphatidylethanolamine in the lipid extracts. Furthermore, D- and L- alanine were quantified after alkaline hydrolysis of the lipid extracts, and only the D enantiomer was found. The authors conclude that D-alanyl phosphatidylglycerol is present, which is a D-alanine carrier from the cytosol to lipoteichoic acid.

*YL:* *We hypothesize that D-alanyl-PG may serve as the intermediate for D-alanylation of wall- and lipo-teichoic acids. We haven’t proved this.*

As already mentioned by referee 1, the most interesting finding of this study is that alanyl phosphatidylglecerol is composed solely of the D enantiomer. However additional control measurements which support this finding are highly recommended. I support the suggestion of referee 1 to test if alanyl-PG is formed in MprF deficient mutants, other controls with chemical standards are possible as well.

*YL:* *We have acquired and studied the lipid composition of the mprF mutant strain. This mprF mutant strain indeed produced alanyl-PG,* *which is consistent to our hypothesis.*

In Figure 8, the ion count of D-alanine in the cell lysate seems much larger as in the lipid lysate; it is difficult to understand how this leads to the same concentration estimate  (0.05 mM). In general the assumption that “the cell has ~10 fold denser d-alanine than membrane” appears speculative and is based on several assumptions (linear response of the instrument, quantitative hydrolysis and extraction). I suggest to remove this part or to make a more elaborate analysis.

*YL: It was an error. Thanks for pointing this out. The correct value of the alanine concentration in the 0.1 ml lipid lysate should be ~0.005 mM. The peak width stayed the same, and the peak high was linear to the concentration of D-alanine derivative. The linear intrapolation was valid.*

Have the authors analysed alanyl PGs in the chloroform rich phase after hydrolysis to check the efficiency of the hydrolysis? Furthermore the authors could measure the concentrations of free alanine by applying the same protocols without adding NH _4_OH to check the efficiency of the hydrolysis step.

*YL: We found that HCl and NaOH at less than 50 mM was compatible with TLC analysis without distorting the lanes. So was 1.0 M of volatile NH4OH, formic acid and acetic acid. Completion of hydrolysis and partitioning of alanine in the aqueous phase were both traced by TLC with ninhydrin staining. The three acidic conditions were inefficient in releasing alanine. NaOH and NH4OH were found to be efficient in releasing alanine. We further chose to use the low concentration of NaOH in lipid hydrolysis for its relative ease to acidify by formic acid, which facilitates free fatty acid partitioning in the chloroform-rich phase.*

The authors reported an irreproducible measurement of aminoacyl PE, potentially caused by the drying process. How long did this 30C drying procedure take? The formation of aminoacyl PEs due to chemical reactions of PE and aminoacyl PGs would indicate an instability of the aminoacyl PGs. Therefore, how reproducible are the measurements of the aminoacyl PGs (variation in ion count)? Are these also influenced by the drying process?

*YL: The drying process typically took a few hours. Unlike aminoacylated PEs, aminoacylated PGs were always detected in ~10 samples with a range of ion counts within 10 fold prior to manuscript preparation. The fluctuation could be partially addressed to constant optimization process which changes experimental parameters such as pH and buffer concentration. Now we have standardized cell culture protocol with 10 mM pH 7 sodium phosphate buffer in the LB media and standardized lipid extraction protocol, the most recent triplicates showed ion counts within 2 fold.*

The authors used collision energies of +45 and -65eV with the QTRAP MS and -40 and +50 eV with the Q-Tof system. Potential caveats resulting from the differently chosen fragmentation settings should be discussed.

*YL: The design of the two system are too different to address the implication of different energies on the fragmentation pattern. As far as we know, the tandem MS spectra were consistent within a 20 eV range with changes in ion counts but not in the overall fragmentation pattern. Tandem MS spectra were acquired at multiple collision energies. Generally, higher collision energy enriches smaller fragments, while lower energy enriches larger fragments. Only the spectra with the most fragments detected with adequate abundance were analysed.*

What is the m/z of the precursor ion for the tandem MS measurements of deprotonated lysyl and alanyl PG (Figure 2)? m/z 821.5 and 792.5?

*YL: The [M-H]- precursor ion of the alanyl-PG had an observed m/z value of 792.5405 amu. Its calculated mass is 792.5394 amu. The precursor lysyl-PG anion had an observed m/z value of 821.5672 amu versus the calculated value of 821.5660 amu. However, figure 2 shows the data acquired with the Sciex 400 QTRAP system which has a lower resolution than the Agilent Q-TOF 6550 system.*

We suggest to remove figure 10, as it provides no relevant information in the context of the findings reported in this study.

*YL: We felt it is somewhat useful to describe the bioinformatics analysis which indicated that DltD is likely a lipase-like enzyme.*

# Peer review: Referee response for version 1

**DOI:** 10.5256/f1000research.8441.r12662

**Published:** 2016-03-09

**Authors:** Zeeshan Ahmed

**Affiliations:** 1 The Jackson Laboratory, Farmington, CT, USA

The manuscript "Profiling and tandem mass spectrometry analysis of aminoacylated phospholipids in *Bacillus subtilis*" reports the contribution of authors in profiling and tandem mass spectrometry analysis of aminoacylated phospholipids in *Bacillus subtilis. *In general manuscript is

- very well written,
- language is good,
- citations are up to date,
- writing is to the point, 
- in scope of the journal,
- justified **Introduction**,
- very well described **Materials and methods**,
- very well presented and discussed **Results**,
- Quality of figure is good and legends are well described,
- Good points raised in **Discussion**,
- Data is provided.

I am personally satisfied with the paper and I would like to congratulate authors of good work. I wanted to mention some points but most of those have been already addressed by the other reviewer (Otto Geiger). I agree with Dr Geiger's publicly available comments, especially:

- Addressing "why *Escherichia coli* is used at all in this study as a reference strain?".
- Minor comments.

I would like to request authors to please address these before final submission.

Thanks.

I have read this submission. I believe that I have an appropriate level of expertise to confirm that it is of an acceptable scientific standard.

# Response

**Published:** 2016-03-14

**Authors:** Yu Luo

**Affiliations:** Department of Biochemistry, University of Saskatchewan, Canada

*YL: Thank you Dr. Ahmed for your comments.*

I am personally satisfied with the paper and I would like to congratulate authors of good work. I wanted to mention some points but most of those have been already addressed by the other reviewer (Otto Geiger). I agree with Dr Geiger's publicly available comments, especially:

- Addressing "why *Escherichia coli* is used at all in this study as a reference strain?".
*YL: This strain was used as the negative reference for its absence of aminoacylated lipids. Indeed its lipid composition was essentially identical to that reported for the K12 strain in reference 24.We will describe this in the revised version.*
***YL: As suggested by Dr. Geiger, we have now acquired and studied the mprF mutant strain of B. subtilis 168. It indeed produced alanyl-PG but not lysyl-PG. We will mention this result in the revised discussion section. Since we are in the process of acquiring and studying several other lipid synthase mutant strains, we will report this piece of experimental data in a future publication.***

# Peer review: Referee response for version 1

**DOI:** 10.5256/f1000research.8441.r12605

**Published:** 2016-02-23

**Authors:** Otto Geiger

**Affiliations:** 1 Centre for Genomic Sciences, National Autonomous University of Mexico, Cuernavaca, Mexico

This manuscript by Atila and Luo reports on the profiling and tandem mass spectrometry analysis of aminoacylated phospholipids in *Bacillus subtilis*. Lipids from *Escherichia coli* and *B. subtilis* are analyzed by thin-layer chromatography and mass spectrometry, but no mass spectral data are shown for *E. coli* lipids or anything that supports the claim that *E.* *coli* lipids were rich in C15:0 fatty acid. Other mass spectral data presented suggest that phosphatidylglycerol (PG) can be substituted with most proteinogenic amino acids, however, lysyl- and alanyl-PG are certainly the most abundant. The authors report extensively (Figs. 4, 5) on lysyl- and alanyl-phosphatidylethanolamine (PE) but come to the conclusion that that these structures were artifacts generated during the isolation procedure. The potentially most interesting finding is that alanyl-PG is almost exclusively substituted with the D-alanyl isomer. In general, the manuscript seems scientifically sound. The mainly mass spectral analysis data make the manuscript somehow descriptive and leave much room for speculation. Instead, one would like to see some complementary data that solidify some of the ideas presented here.

Major Comments:

It is not exactly clear why *Escherichia coli* is used at all in this study. – As a reference strain? If so, why then a strain that is used for expression of genes? What really surprises me is that the authors find that “Lipids from the *E. coli* strain were richest in saturated pentadecanoic acid (C15:0) and …”. - Whatever previous references you consult, C15:0 is never mentioned as a major fatty acyl residue in *E. coli*. For example, see Mejía *et al. *(1999). If it is true that C15:0 is a major fatty acid in *E. coli*, this finding would certainly put in doubt some dogmas. 

Membrane lipid biosynthesis and composition of *Bacillus subtilis* strain 168 has been studied by numerous groups to a considerable extent. One of the major results of the present paper is that alanyl-PG carries exclusively the D-alanyl form and the authors discussed extensively that D-alanyl-PG should be formed by a mprF-independent pathway. There are MprF-deficient mutants available from John Helman´s group (Salzberg and Helman, 2008) and if the authors are correct, one would expect that these mutants still can make D-alanyl-PG. 

Fig. Legend 8: Mention here or somewhere that the “340 amu molecular anion” corresponds to alanine + Marley´s ?

I don´t understand the last part of the Discussion “with overall similarity to platelet-activating factor”. Platelet-activating factor is a relatively hydrophilic lipid; so which similarity can it have to a protein? 

Minor Comments:

1. Page 2, second paragraph of Introduction: change “peptitoglycan” to “peptidoglycan”
2. Page 3, left column, third paragraph, and elsewhere: The symbol for “micro” used here is an “u” not the Greek symbol as it should be.
3. Table 1: change “Calculated Msss” to “Calculated mass”.

I have read this submission. I believe that I have an appropriate level of expertise to confirm that it is of an acceptable scientific standard, however I have significant reservations, as outlined above.

## References

[1] MejíaRGómez-EichelmannMCFernándezMS: Fatty acid profile of Escherichia coli during the heat-shock response. *Biochem Mol Biol Int*.1999;47(5) :835-44 1036525410.1080/15216549900201923

[2] SalzbergLIHelmannJD: Phenotypic and transcriptomic characterization of Bacillus subtilis mutants with grossly altered membrane composition. *J Bacteriol*.2008;190(23) : 10.1128/JB.00720-08 7797-807 10.1128/JB.00720-08 18820022PMC2583605

# Response

**Published:** 2016-03-02

**Authors:** Yu Luo

**Affiliations:** Department of Biochemistry, University of Saskatchewan, Canada

This manuscript by Atila and Luo reports on the profiling and tandem mass spectrometry analysis of aminoacylated phospholipids in *Bacillus subtilis*. Lipids from *Escherichia coli* and *B. subtilis* are analyzed by thin-layer chromatography and mass spectrometry, but no mass spectral data are shown for *E. coli* lipids or anything that supports the claim that *E.* *coli* lipids were rich in C15:0 fatty acid. Other mass spectral data presented suggest that phosphatidylglycerol (PG) can be substituted with most proteinogenic amino acids, however, lysyl- and alanyl-PG are certainly the most abundant. The authors report extensively (Figs. 4, 5) on lysyl- and alanyl-phosphatidylethanolamine (PE) but come to the conclusion that that these structures were artifacts generated during the isolation procedure. The potentially most interesting finding is that alanyl-PG is almost exclusively substituted with the D-alanyl isomer. In general, the manuscript seems scientifically sound. The mainly mass spectral analysis data make the manuscript somehow descriptive and leave much room for speculation. Instead, one would like to see some complementary data that solidify some of the ideas presented here.

***YL:* We appreciate Dr. Otto Geiger's comments and suggestions. Our response as listed below and clarification will be incorporated in the revised version. It was a fact, perhaps an unfortunate distraction, that we had spent a lot of time on aminoacylated PE.**

Major Comments:

It is not exactly clear why *Escherichia coli* is used at all in this study. – As a reference strain? If so, why then a strain that is used for expression of genes?

***YL: This strain is first used as a reference strain which is not expected to produce aminoacylated PG. It is also planned to be used in the near future as the host for expressing DltABCD proteins.***

What really surprises me is that the authors find that “Lipids from the *E. coli* strain were richest in saturated pentadecanoic acid (C15:0) and …”. - Whatever previous references you consult, C15:0 is never mentioned as a major fatty acyl residue in *E. coli*. For example, see Mejía *et al. *(1999). If it is true that C15:0 is a major fatty acid in *E. coli*, this finding would certainly put in doubt some dogmas. 

***YL: The mass spectra of the E. coli strain were similar to that reported in reference 24 with (33:1) PG and PE being the most abundant. It was richest in (16:0) and (14:0) saturated fatty acids along with (17:1) and (18:1) monounsaturated or cyclopropane fatty acids. There was a less abundant 241 amu fragment ion matching deprotonated (15:0) saturated fatty acid or (14:0) epoxy fatty acid. However, it was not the aim of this study to characterize the fatty acid composition.***

Membrane lipid biosynthesis and composition of *Bacillus subtilis* strain 168 has been studied by numerous groups to a considerable extent. One of the major results of the present paper is that alanyl-PG carries exclusively the D-alanyl form and the authors discussed extensively that D-alanyl-PG should be formed by a mprF-independent pathway. There are MprF-deficient mutants available from John Helman´s group (Salzberg and Helman, 2008) and if the authors are correct, one would expect that these mutants still can make D-alanyl-PG.

***YL: We have a plan to gather mutants of Bacillus subtilis strain 168, which are relevant to lipid biosynthesis. Since MprF's substrate is L-aminoacylated tRNA, we reasoned that the overwhelming abundance of D- over L-alanine in the lipid lysate implied that an mprF-dependent pathway likely produced D-alanyl-PG. During the past week, the MprF-deficient strain has been acquired as suggested and its lipid extract studied. As expected, the lipid did not contain lysyl-PG. Importantly, it did contain alanyl-PG, which is consistent to our hypothesis.***

Fig. Legend 8: Mention here or somewhere that the “340 amu molecular anion” corresponds to alanine + Marfey´s ?

***YL: We will add a statement that the 340 amu molecular anion corresponds to alanine derivatized by Marfey’s reagent.***

I don´t understand the last part of the Discussion “with overall similarity to platelet-activating factor”. Platelet-activating factor is a relatively hydrophilic lipid; so which similarity can it have to a protein? 

***YL: We are sorry to have missed the word “acetylhydrolase”. The remote homolog is platelet-activating factor acetylhydrolase. ProFunc server found a possible match with an E-score of 0.078 to a platelet-activating factor acetylhydrolase (PDB entry 1BWR). A hydrogen-bonded triad of Ser-47 / Asp376 / His 379 corresponding to the active site of this acetylhydrolase was found conserved in the DltD structure.***

Minor Comments:

1.     Page 2, second paragraph of Introduction: change “peptitoglycan” to “peptidoglycan”

2.     Page 3, left column, third paragraph, and elsewhere: The symbol for “micro” used here is an “u” not the Greek symbol as it should be.

3.     Table 1: change “Calculated Msss” to “Calculated mass”.

***YL: We will correct these errors in the revised version.***

**References**

1. Mejía R, Gómez-Eichelmann MC, Fernández MS: Fatty acid profile of Escherichia coli during the heat-shock response. *Biochem Mol Biol Int*. 1999; **47** (5): 835-44 PubMed Abstract

***YL: We appreciate this reference.***

 .

2. Salzberg LI, Helmann JD: Phenotypic and transcriptomic characterization of Bacillus subtilis mutants with grossly altered membrane composition. *J Bacteriol*. 2008; **190** (23): 7797-807 PubMed Abstract | Publisher Full Text 

***YL: We plan to request all the mutant strains produced by the authors of this article. We have since acquired and studied the pssA and mprF mutants.***
